# Balancing Inflammation and Central Nervous System Homeostasis: T Cell Receptor Signaling in Antiviral Brain T_RM_ Formation and Function

**DOI:** 10.3389/fimmu.2020.624144

**Published:** 2021-01-27

**Authors:** Colleen S. Netherby-Winslow, Katelyn N. Ayers, Aron E. Lukacher

**Affiliations:** Department of Microbiology and Immunology, Penn State College of Medicine, Hershey, PA, United States

**Keywords:** T cell receptor, PD-1, brain-resident memory CD8 T cells, virus, neuroinflammation

## Abstract

Tissue-resident memory (T_RM_) CD8 T cells provide early frontline defense against regional pathogen reencounter. CD8 T_RM_ are predominantly parked in nonlymphoid tissues and do not circulate. In addition to this anatomic difference, T_RM_ are transcriptionally and phenotypically distinct from central-memory T cells (T_CM_) and effector-memory T cells (T_EM_). Moreover, T_RM_ differ phenotypically, functionally, and transcriptionally across barrier tissues (e.g., gastrointestinal tract, respiratory tract, urogenital tract, and skin) and in non-barrier organs (e.g., brain, liver, kidney). In the brain, T_RM_ are governed by a contextual milieu that balances T_RM_ activation and preservation of essential post-mitotic neurons. Factors contributing to the development and maintenance of brain T_RM_, of which T cell receptor (TCR) signal strength and duration is a central determinant, vary depending on the infectious agent and modulation of TCR signaling by inhibitory markers that quell potentially pathogenic inflammation. This review will explore our current understanding of the context-dependent factors that drive the acquisition of brain (b)T_RM_ phenotype and function, and discuss the contribution of T_RM_ to promoting protective immune responses *in situ* while maintaining tissue homeostasis.

## Introduction

Development of long-lived T cell memory is vital to protection against microbial pathogens and cancer, and a goal of vaccination efforts. Initial work identified T_CM_ which, like naive T cells, survey secondary lymphoid organs, and T_EM_, which circulate in the blood and non-lymphoid tissues. Because of their increased numbers over naïve T cell precursors to a particular antigen, and their lower threshold for activation and reduced dependence on costimulation, T_CM_ and T_EM_ respond rapidly to pathogen reencounter (1, 2). Nearly 20 years ago, evidence emerged supporting the idea that a population of memory T cells poised with an effector arsenal resided in non-lymphoid tissues (3). More recent evidence suggests that T_RM_, like T_CM_, are derived from a common naive T cell precursor after local antigen exposure (4). While sharing many effector capabilities with T_EM_, T_RM_ differed from T_EM_ in expression of trafficking molecules and having a distinct gene expression signature (5). The classification of T_RM_ as a separate subset of CD8 T cell memory prompted new investigations to define the factors that contribute to T_RM_ development and maintenance, how T_RM_-mediated immunity contributes to the dynamic immune response to microbial pathogens, and if T_RM_ function can be harnessed for a multimodal therapeutic approach to treat or prevent infection and cancer.

An additional layer of complexity is that T_RM_ are not a homogeneous subset, because tissue environments themselves impose tissue-specific heterogeneity to T_RM_. Most T_RM_ characterization has been done in barrier tissues; far less is understood how T_RM_ establish themselves in non-barrier sites. In particular, the brain and spinal cord are especially sensitive to tissue injury and loss from pro-inflammatory mediators. Mouse models of CNS infection, including by vesicular stomatitis virus (VSV), lymphocytic choriomeningitis virus (LCMV), *Toxoplasma gondii*, murine cytomegalovirus (MCMV), and mouse polyomavirus (MuPyV), have identified T_RM_ in the brain that confer antigen-specific protection against reinfection (5–9). It is likely that brain-specific factors contribute to formation of T_RM_ and their functional attributes due to the exquisite need to balance immune activation and tissue preservation in the CNS.

The trajectory of T cell differentiation is initiated by TCR engagement, then modified by costimulation and inflammation (10). The integration and duration of these signals directs a naïve T cell toward effector or memory fates, with peptide:MHC (pMHC) ligand-TCR interaction being the critical first step that guides the memory response. The strength of signal transduction events orchestrated after TCR binding with its cognate pMHC regulates induction of transcription factors, tissue-trafficking adhesion molecules, and cytokine receptors required for T_RM_ generation. Thus, TCR signal strength per se dictates the quality and abundance of the resulting T_RM_ population (11, 12). Additionally, regulating TCR signaling *via* inhibitory receptors, such as programmed cell death protein-1 [PD-1(CD279)], may be essential for T_RM_ maintenance in particular tissues by operating as a rheostat to fine tune T cell activation and effector function. This review will focus on how TCR signaling shapes the T_RM_ pool and how inhibitory receptor signaling drives the balance between effector function and long-term maintenance in tissues, an issue of especial importance in the CNS.

## T_RM_ Identification in Barrier vs. Brain Tissue

**Figure 1:**
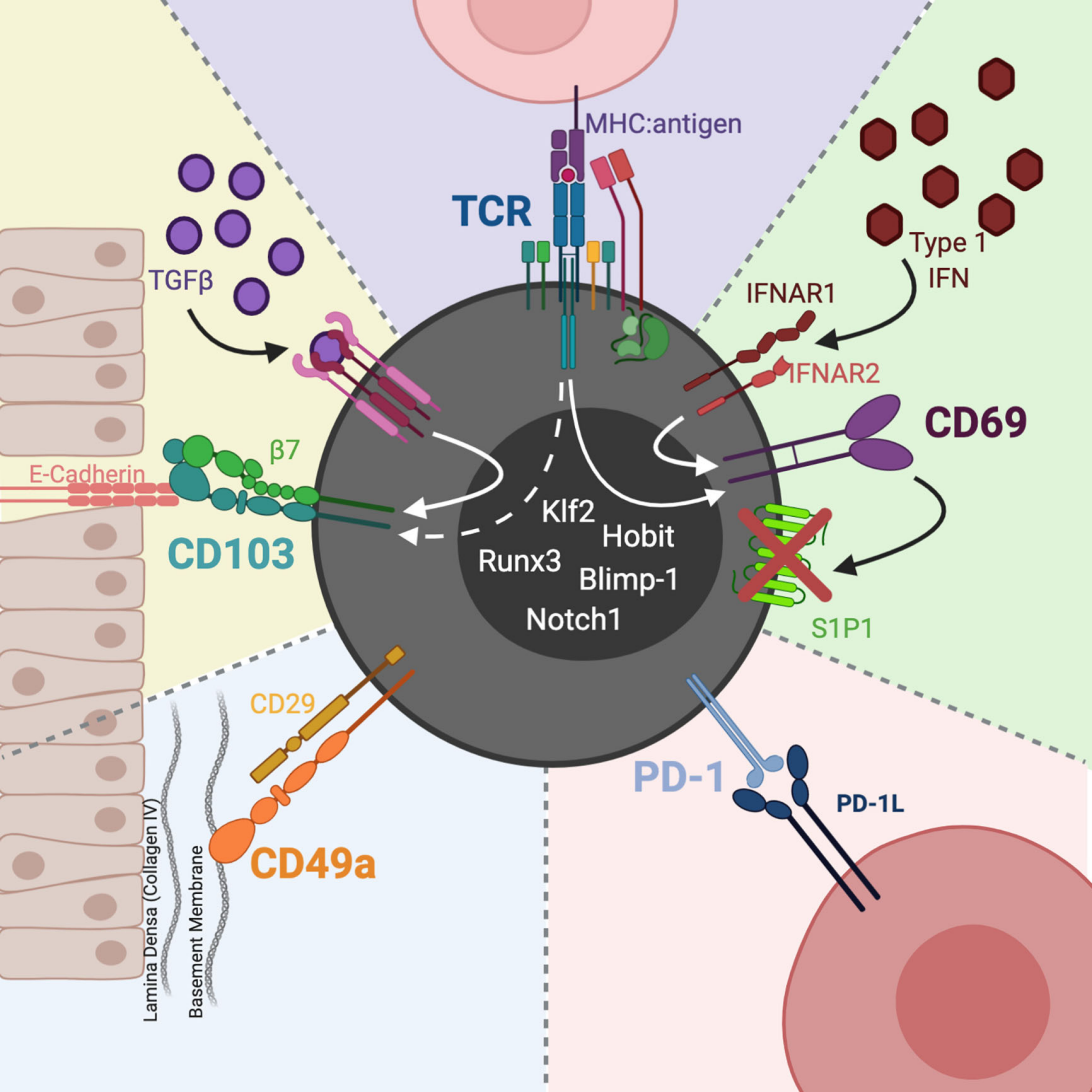
CD8 T_RM_ phenotype and heterogeneity. CD103 is the receptor for the epithelial junction protein, E-cadherin. The CD103:E-cadherin interaction moors the T cell to the epithelial mucosa. TGFβ induces expression of CD103, whose levels may also be affected by TCR activation. CD49a partners with CD29 (integrin β1) to constitute the heterodimer VLA-1. VLA-1 binds collagen, with a predilection for Col IV in epithelial basement membranes. CD69 is a C-type lectin upregulated by type 1 IFNs as well as TCR activation. Once expressed, CD69 hinders T_RM_ egress by complexing with S1P1, leading to S1P1s internalization and degradation. In particular sites, such as the CNS, T_RM_ express PD-1 which acts to maintain functional T_RM_ and preserve tissue homeostasis. Downregulation of Klf2 and upregulation of Blimp-1, Runx3, Notch1, and Hobit transcription factors have also been used to define T_RM_. Image created with BioRender.com.

T_RM_ are distinguished from circulating memory T cells by the expression of the integrins CD103 (αE subunit of the αEβ7 heterodimer) and CD49a (alpha subunit of the CD49a/CD29 heterodimer), as well as the C-type lectin CD69; these molecules act to direct and retain T cells in tissues (**Figure 1**). Additionally, T_RM_ are phenotyped by the absence of cell surface sphingosine-1-phosphate receptor 1 (S1P1), the CCR7 chemokine receptor, and CD62L (L-selectin); these molecules contribute to T cell homing to (CCR7, CD62L) and egress from (S1P1) lymph nodes (13). The activating transcription factor Kruppel-like factor 2 (Klf2) targets the S1P1 gene and Klf2 downregulation is also used to define T_RM_ (14). CD103 is a common marker for T_RM_ due to its association with epithelial localization and tissue retention (15), but the requirements for CD103 expression for T_RM_ development or maintenance is a topic of some debate (16).

A role for CD103 integrins in T_RM_ retention in epithelial sites, like skin, lungs, salivary glands, and intestinal and female reproductive tract mucosa makes intuitive sense, due to its binding to the epithelial junction protein, E-cadherin. CD103 expressing T cells, however, can also be found in locations distant from epithelium, such as the brain and other non-barrier tissues; the function of CD103 expressed by T_RM_ in these locations is unclear. Using peripheral infection and dendritic cell-mediated immunization, Urban et al. recently demonstrated that non-CNS infections generated CD8 bT_RM_. Notably, few of these CD8 bT_RM_ expressed CD103 and donor CD103^−/−^ CD8 T cells yielded CD8 bT_RM_ at the same levels as donor WT cells (17). These data indicate that CD103 is dispensable for generating CD8 bT_RM_, which contrasts with the apparent requirement for CD103 for establishment of intestinal CD8 T_RM_ (18).

To this point, CD103^-^ T_RM_ in the brain retain T_RM_ migratory and phenotypic properties (e.g., being tissue-sessile, CD69^+^, and CD49a^+^) as well as T_RM_ gene expression signatures (19). During persistent infection with MuPyV, a natural mouse pathogen, CD103^+^ bT_RM_ are more efficient effectors (7), which is consistent with evidence of signaling from CD103:E-cadherin interactions enhancing CD8 T_RM_ function, cytoskeleton reorganization, migration, cytokine release, and cytotoxicity (20–22). Although members of the cadherin family have been implicated in regulating neuron synaptic plasticity and flow cytometric analysis has shown E-cadherin expression on certain immune cells like dendritic cells and even some T_RM_ (23–27), E-cadherin is predominantly expressed in epithelial tissues. With regard to CD103^+^ CD8 bT_RM_, however, there is little published data on E-cadherin expression in the brain, but it has been proposed that perhaps CD103^+^ brain CD8 T cells are interacting with E-cadherin-expressing immune cells rather than epithelial cells (16, 28). Aberrant expression of E-cadherin has also been associated with a more aggressive tumor subtype (28), but whether chronic inflammation or cancer alters E-cadherin expression in neural tissue is an open question. Alternatively, another ligand in the CNS may bind CD103 integrins expressed by CD8 bT_RM_. TGFβ is a well-documented inducer of CD103 on T_RM_ (18). TGFβ receptor signaling acting concomitantly with TCR stimulation may modulate CD103 expression levels. This possibility raises the broader issue of whether TGFβ and pMHC availability act together or independently to affect T_RM_ development, location, and function. Although CD103 expression seems to be specific to T_RM_, it is variably expressed by T_RM_ in different tissues and is arguably dispensable for T_RM_ functions. For example, CD103 blocking antibody does not negate the ability of lung CD8 T_RM_ to protect mice from lethal influenza infection (29). Thus, the requirements for CD103 for CD8 bT_RM_ maintenance, and the precise role TCR signaling plays in regulating CD103 expression warrants investigation. CD49a’s role in T_RM_ development is less well defined than for CD103. CD49a does not directly attach to epithelia like CD103, but collagen IV (ColIV), its primary ligand, is positioned in the lamina densa layers of epithelial basement membranes (16, 30). The CD49a:ColIV interaction could then result in T_RM_ localization to the epithelium and subsequent tethering to CD103:E-cadherin. Furthermore, in influenza infection CD49a protects lung CD8 T_RM_ from apoptosis in part *via* interactions with collagen IV (31). A recent study shows that CD49a is required for T_RM_-mediated protection from lethal influenza pulmonary infection (29). In the skin, however, CD49a seems to influence the effector function of T_RM_, with CD49a^+^ CD8 T_RM_ producing IFN-λ and CD8^+^ CD49a^-^ T_RM_ producing interleukin (IL)-17 (32). Although CD69 is often used as a marker of recent T cell activation, it is expressed by T_RM_ in most tissues including those of the CNS (33). CD69 is also upregulated by type I interferons independent of TCR engagement (34). CD69 binds to and induces degradation of S1P1, which enables T cells to migrate along sphingosine-1-phosphate (S1P) gradients (SIP is higher in lymphatics than tissues). The expression profile for CD69, CD103, and CD49a, however, is not exclusive to nor is it uniform across T_RM_; disappointingly, there is no cleanly defined T_RM_ phenotype (15).

Identifying T_RM_ is made more challenging by evidence that T_RM_ can be phenotypically heterogeneous even in the same organ (15). In mice intracranially (i.c.) inoculation with an attenuated LCMV variant, only ~50% of the bT_RM_ are CD103^+^ (9). During persistent infection with MuPyV, the vast majority of virus-specific CD8 T cells in the brain are CD69^+^, but only ~40% expressed CD103 (19). In addition, the fraction of CD103^-^ cells co-expressing CXCR5^hi^ and TCF-1^hi^ cells was higher than the CD103^+^ subpopulation. Elevation of both the transcription factor TCF-1 and the chemokine receptor CXCR5 on memory CD8 T cells has been linked to increased functional capability during chronic infection (35). This is noteworthy since in chronic viral infections TCF-1 and CXCR5 aid in establishing a population of proliferation-competent memory CD8 T cell precursors to maintain a pipeline leading to end-stage exhausted T cells (T_EX_) (36). The CD103^+^ and CD103^-^ subsets, interestingly, expressed similar levels of Ki67 expression and antigen-stimulation IFN-γ production, indicating comparable proliferative and functional capabilities, respectively; however, the CD103^+^ subpopulation displayed higher effector activity (7, 19). A strategy to help reconcile these apparent discrepancies is to further stratify T_RM_ by overlaying expression of additional transcriptome molecules and cytokine receptors linked to T_RM_ differentiation, including Runx3, Notch, Hobit, and Blimp-1, as well as the receptors for IL-15, Type I IFN, TGF-β, and IL-12 (13, 37). Due to the phenotypic heterogeneity across T_RM_ populations and shared markers with other CD8 T cell subsets, more in-depth “clustering” of these molecules may help not only to ensure that a T cell is a bona fide T_RM_ but also to uncover additional breadth of T_RM_ diversity between and within tissues.

An under-appreciated feature of T_RM_ cells is the upregulation and maintenance of immune checkpoint molecules, particularly PD-1, in certain tissues and with particular viral infections (19, 38). T_RM_ generated in the skin after HSV-1 infection or the brain following MuPyV infection have increased surface expression of multiple inhibitory receptors in addition to PD-1, but retain at least partial functionality (7, 39). PD-1 is transiently expressed by CD8 effector T cells after antigen receptor signaling, but even here PD-1 inhibits functionality (40). The appellation “persistent infection” as a catchall belies the complexity of lifecycles by viruses that co-reside long-term with their hosts, such as latency-reactivation by herpesviruses vs. smoldering infections by papillomavirus and polyomaviruses. Whether bona fide memory T cells develop in the setting of persistent infection is often debated. Often overlooked, however, is the nature of the persistent infection, which depending on level, location, and timing of epitope availability may allow co-habitation by both memory and effector T cells. Compounding this complexity is that some viruses previously thought to be completely cleared after acute infection (e.g., influenza, VSV) leave residual T cell epitope-bearing antigen-presenting cells (APCs) for several weeks (41–43). Unremitting strong TCR stimulation in neoplasia and chronic viremia arguably should be considered separately from transient/low-level persistent viral infections, as the former typically render CD8 T cells profoundly dysfunctional and direct them toward an adaptive state of differentiation termed T_EX_ (44). Yet, even under these circumstances T_EX_ exert antiviral activity as evidenced by the outgrowth of CD8 T cell epitope escape variants in HIV infection (45, 46). Although PD-1, as well as CTLA-4 and TIM-3, are upregulated and sustained on the surface of CD8 T cells infiltrating tumors and in chronically infected tissues, these T cells can express molecules and gene signatures shared with T_RM_ (47, 48). Similar to its role in checking T cell-mediated autoimmunity, checkpoint inhibitors mitigate T cell-mediated immunopathology (19, 38, 49, 50). PD-1 expression as well as its role in the cell’s functional adaptivity may distinguish T_RM_ from other memory CD8 T cell subsets that infiltrate the CNS (19, 38, 48).

## TCR Signal Strength as a Driver of t_rm_ Fate and Function

TCR signaling has been implicated in the formation of a diverse memory pool. From its initial description in the early 1980s (51), extensive research has been conducted on how signals induced when the TCR engages the pMHC complex directs effector memory differentiation and function. The relative “strength” of the TCR signal is the composite of affinity of the pMHC ligand for its cognate TCR, the amount of antigen presented on the surface of the APC (i.e., pMHC epitope density), the number of cell surface TCRs, and the duration of the TCR:pMHC interactions (52–54). The prevailing model holds that activation through the TCR orchestrates an instructional program that directs CD8 T cell expansion, effector differentiation, contraction and memory formation (55). In addition, co-stimulation through CD28, CD27, CD40, 4-1BB, and/or ICOS during priming of naïve T cells further tailors T cell fate (56–60). Cytokine input complements TCR activation to select differentiation programs and T cell longevity. For example, IL-12 promotes effector function and survival (61, 62), and IL-15 supports homeostatic maintenance of memory T cells (63–65). Kaech and colleagues have shown that a critical determinant whether a naive T cell becomes a short-lived effector cell (SLEC) or a memory precursor effector cell (MPEC) is the amount of IL-12 present during naïve T cell priming (66). IL-12 was found to regulate the level of expression of the T-box transcription factor T-bet (Tbx21) in a dose-dependent manner; high levels of T-bet instructed cells to become SLECs, and low T-bet expression favored MPEC development. Together with strength of TCR signaling, a complex tapestry of inflammatory signals and co-stimulation coalesce to influence the size and durability of a T cell memory response.

TCR signal strength also quantitatively and qualitatively shapes memory T cell differentiation. Disruption in TCR proximal signaling *in vivo* by mutating SLP-76 caused impaired Ca^2+^ influx and dampened T cell activation, without disrupting the expansion of CD8 T cells in response to acute LCMV infection (67). Weaker TCR stimulation in SLP-76 mutant mice biased CD8 T cells toward memory differentiation, with weak TCR stimulation favoring the production of cells with a CD62L^hi^ T_CM_ phenotype. Our group found that CD8 bT_RM_ generated during persistent MuPyV infection possess high-affinity TCRs compared to counterparts in the spleen and kidney. Because virus-specific CD8 T_EFF_ also express high-affinity TCRs, we suggested that these cells were the progeny of high-affinity effectors recruited to the brain during the acute stage of infection (68). Indeed, we observed that there is a window of opportunity for immune cells, and possibly virus, to breach a blood-brain barrier rendered permeable during acute MuPyV encephalitis (69). A plausible possibility is that high-affinity TCRs enable CD8 bT_RM_ to detect low levels of viral antigen during persistent infection (68).

During MuPyV infection, our group reported that weaker TCR stimulation favored expansion of CD8 bT_RM_ having superior ability to respond to homologous MuPyV i.c. re-infection (11). Using site-directed mutagenesis to alter a subdominant epitope in a nonstructural viral protein of MuPyV, Maru et al. generated a panel of viruses with non-synonymous mutations in a CD8 T cell epitope to assess *in vivo* the impact of TCR stimulation strength per se on bT_RM_ differentiation. By using adoptively transferred CD8 T cells from a TCR transgenic mouse recognizing a subdominant epitope, these authors controlled the size, recruitment, and clonality of the naïve T cell response, and circumvented the confounding problems of changes in virus levels and inflammation over the course of infection. Although CD8 bT_RM_ generated in a setting of suboptimal TCR stimulation enjoyed a more robust ability to expand upon pathogen reencounter, no impact on effector function was observed. Similarly, Langlois and colleagues reported an advantage in forming influenza-specific lung CD8 T_RM_ after stimulation with low-affinity epitopes (12). Here, TCR transgenic OT-I CD8 T cells (specific for the H-2K^b^-restricted SIINFEKL peptide from chicken ovalbumin residues 257–264) were adoptively transferred to mice infected with a recombinant influenza virus encoding native and altered OT-I epitopes. Although high- and low-affinity stimulated OT-I T_RM_ had similar phenotype and function, transcriptional profiling revealed that T_RM_ generated by low-affinity stimulation expressed increased pro-survival factors, which would favor long-term maintenance in tissues. CD8 bT_RM_ having high-affinity TCRs would likely be selected by suboptimal TCR stimulation allowing them to engage low-density epitopes or epitopes modified to limit binding to TCRs (70). The level and duration of TCR stimulation, in concert with tissue-specific cytokines, may result in upregulation of inhibitory receptors on CD8 T_RM_ to modulate their TCR signal strength, and thereby control their effector capabilities and survival (7, 71).

## The Need to Regulate TCR Signal Strength in bT_RM_

Unchecked T cell activation can cause autoimmunity and immunopathology. To prevent this, inhibitory receptors constrain T cell effector functions and proliferation following TCR engagement and are upregulated in chronic infection and cancer, with the level of expression and number of inhibitory receptors dictated by the density and duration of cognate epitope (72). The importance of PD-1 and other inhibitory receptors in mitigating T cell function and prolonging longevity are well-established in animal models and humans, where blockade of PD-1 or PD-L1 reinvigorates T cell responses, reduces viral load, and/or boosts tumor control. PD-1 primarily regulates T cell activity by dampening intracellular stimulatory signals from the TCR/CD3 complex. When the PD-1 monomeric receptor engages its ligands PD-L1 (CD274)/PD-L2 (CD273), its cytoplasmic immunoreceptor tyrosine-based inhibition motif (ITIM) and immunoreceptor tyrosine-based switch motif (ITSM) domains are phosphorylated, resulting in binding by the Src homology 2 domain-containing phosphatase 2 (SHP2) (73). Subsequent SHP2 activation leads to tyrosine dephosphorylation of signaling molecules downstream of TCR and costimulatory receptors (74). PD-1 signaling can also result in metabolic reprograming; e.g., PD-1 signaling reduces Akt activity, suppressing mTOR (75). This effectively switches T cell metabolism from glycolysis to fatty acid oxidation (FAO). T_RM_ have a dynamic metabolic profile, but predominantly utilize oxidative phosphorylation (76). Skin CD8 T_RM_ make use of exogenous fatty acids for FAO (77). Whether CD8 bT_RM_ share this metabolic pathway remains to be determined.

PD-1 expression by CD8 T_RM_ appears to be dependent on the tissue environment and the nature of the viral infection. What governs the stability of PD-1 expression and its role in T_RM_ function and maintenance is an area of active interest. In VSV infection, CD8 bT_RM_ express low levels of PD-1 transcripts but no detectable PD-1 protein, whereas bT_RM_ from mice infected with mouse cytomegalovirus (MCMV) or MuPyV are PD-1^hi^ (5, 6, 19, 78). Youngblood et al. established that the PD-1 promoter is dynamically epigenetically regulated, with the extent of demethylation of the PD-1 promoter correlating with the strength and duration of TCR stimulation. During acute LCMV infection, the PD-1 promoter is extensively demethylated and then remethylated upon viral clearance. During chronic LCMV infection, the PD-1 promoter remains demethylated in viral antigen-specific CD8 T cells (79). In MuPyV encephalitis, the PD-1 promoter is likewise heavily demethylated in bT_RM,_ and undergoes only a partial remethylation in virus-specific T cells in the spleen (19). Interestingly, maintenance of PD-1 expression on MuPyV-specific CD8 bT_RM_ was found to be independent of cognate antigen or inflammation (19). In contrast, PD-1^hi^ CD8 T_RM_ in the lungs of influenza-infected mice are maintained by MHC class I signaling and CD80 and CD86 costimulation (80). PD-1 may serve to dampen the level of TCR signaling in CD8 bT_RM_, allowing them to exert some antiviral activity and avoid apoptosis.

Because antigen is required for CD8 bT_RM_ formation but not PD-1 maintenance, it is possible that PD-1 is an important regulator of T_RM_ function specifically in the brain microenvironment. Memory CD8 T cells in the eye, an immune privileged organ, also express PD-1 (81). In a mouse model of coronavirus CNS infection, PD-1 expression on CD8 T cells limits immune pathology and axonal damage (82, 83). The concept that PD-1 expression plays an important regulatory role in the brain is strengthened by evidence that splenic CD8 T_RM_ lack PD-1 expression during persistent MuPyV infection and that PD-L1 blockade limits CD8 bT_RM_ effector function. bT_RM_ produce IFN-γ, which regulates microglial function (84). It is also possible that microglia in turn regulate T_RM_ homeostasis through PD-1:PD-L1 interaction. A complete understanding how PD-1 regulates deleterious CD8 bT_RM_ activation in the setting of persistent viral encephalitides or whether PD-1 may selectively inhibit neuropathological effector activities remains unclear.

## PD-1: an Arbiter of Neuroprotection

CD8 T cells expressing a T_RM_ phenotype (CD69, CD103) and PD-1 progressively accumulate in the brain parenchyma with aging. Cerebral ischemia promotes production of inflammatory mediators by these CD8 bT_RM_ (85). Clonally expanded CD8 T cells with gene signatures for cytokine-producing effector memory cells expressing CD69 and VLA-1/-4 transcripts accumulate in the subventricular zone (SVZ) of aged brains, a neurogenic niche containing neural stem cells (NSC), neural progenitor cells (NPC) and microglia; notably, IFN-γ secreted by CD8 T cells inhibits proliferation of NSCs and NPCs (86). In MuPyV encephalitis, virus-specific CD8 T cells aggregate in the SVZ subjacent to infected ependyma and produce IFN-γ *in situ* (69, 87). It is tempting to speculate that SVZ-localized antiviral CD8 bT_RM_ produce IFN-γ, which is deleterious to neurogenic niches and contributes to cognitive decline in survivors of the life-threatening brain demyelinating disease progressive multifocal leukoencephalopathy (PML) caused by the JC polyomavirus (JCPyV). Following recovery from neuropathic flavivirus infection, IFN-γ from CD8 bT_RM_ has also been show to drive microglia to eliminate synapses in the hippocampus and cause spatial-learning defects (84). These findings raise the ominous spectre that activation of JCPyV-specific CD8 bT_RM_ after PD-1 blockade may compromise learning and memory in PML survivors.

Although PD-1 is highly expressed by CD8 bT_RM_ during encephalitis by MuPyV and MCMV (7, 19, 88, 89), these bT_RM_ do not display a clear exhaustion profile (19, 90, 91). Rather, PD-1 appears to operate in the brain primarily to balance bystander- and virus-induced inflammation and tissue damage against virus control by antiviral bT_RM_ cells (90, 91). In the pancreas, PD-1 ligand-expressing macrophages control the function of the PD-1^+^ CD8 T_RM_ cells. PD-1 blockade of pancreatic CD8 T_RM_ cells significantly augmented their ability to produce IFN-α, TNF-α, and IL-2 upon TCR stimulation (90). In the lung, PD-L1 blockade promoted the expansion of T_RM_ and enhanced secondary protection to influenza infection, but also resulted in the development of inflammation-induced fibrotic injury (80). These results are mirrored in the brain. bT_RM_ in MuPyV-infected PD-L1^−/−^ mice had a higher frequency of IFN-γ-producing cells than bT_RM_ from MuPyV-infected wild type (WT) mice (91). Furthermore, PD-1:PD-L1 interactions were found to quell inflammation in the pancreas and brain (90, 91). CD8 T_RM_ are detected in brains of patients dying of non-neurological causes. Interestingly, these T_RM_ are CD103^+^ CD69^+^ and highly express PD-1 and CTLA-4 (92). bT_RM_ in healthy human brains may be telltale signs of long-resolved infections. These bT_RM_ may also provide the “fertile field” for CNS autoimmune diseases, such as multiple sclerosis by secreting chemokines that attract circulating self-reactive T cells (93). Thus, expression of checkpoint inhibitory receptors, such as PD-1, may act to halt production of such chemokines and the potential for CNS autoimmune diseases. PD-L1 expression by MHC-I/II-expressing CNS-resident cells (e.g., microglia) may, in turn, be critical determinants of susceptibility to CNS autoimmunity. Collectively, these data support the likelihood that CD8 T_RM_ in the brain retain expression of checkpoint inhibitory molecules to limit tissue-injurious inflammation and preserve CNS integrity.

With the heightened effector functionality of T_RM_ consequent to interrupting PD-1 signaling, PD-1 or PD-L1 blockade could be anticipated to enhance T_RM_ response against persistently infecting viral pathogens. In a small randomized and placebo-controlled study, 3 out of 6 patients with hepatitis C virus given a new humanized ligand-blocking PD-1 antibody exhibited 4-log reductions in viral load, but this was associated with immunologic adverse events, including autoimmune thyroiditis (94). In a phase Ib study of patients with chronic hepatitis B virus (HBV) infection, nearly all of the patients given a single infusion of the PD-1 blocking antibody nivolumab experienced a decrease in HBV surface antigen (HBsAg) titers (95). Finally, in individuals with PML, a significant number of patients receiving anti-PD-1 had fewer cerebrospinal fluid JCPyV genome copies, elevated JCPyV-specific CD4 and CD8 T cell responses, and importantly, clinical improvement or disease stabilization (96, 97). A likely critical variable in the success of PD-1 blockade therapy is the severity of infection at the time of therapy initiation, with higher viral burden being associated with greater risk of immune-mediated complications. Although these studies do not directly assign effects of the PD-1:PD-1L blockade to bT_RM,_ they demonstrate the importance of checkpoint inhibitor blockade as an anti-viral therapy in humans. Knowing that bT_RM_ have increased effectivity in mouse models lacking either PD-1 or PD-L1, a plausible hypothesis is that the antiviral effects of the PD-1:PD-1L blockade in humans could be due to resurrected effector activity by bT_RM_.

Beyond affecting the functional capabilities of T_RM_ cells, recent reports suggest that PD-1 is involved in the development of T_RM_ in different tissues, including those in the CNS. During MCMV infection, CD103^+^ CD69^+^ bT_RM_ populations were sparse in PD-L1^−/−^ and PD-1^−/−^ mice compared to WT mice, implicating PD-1 signaling as a positive factor in development of bT_RM_ (89). PD-1 is involved in governing T cell activation, fate, function, and tolerance as well as immune homeostasis (98). Therefore, using a global PD-1 knock-out system could have altered the fate of all T cell subsets and not just that of the bT_RM._ Conversely, in response to MuPyV, a higher frequency of CD103^+^ CD8 T cells populations were observed in brains of PD-L1^−/−^ mice as well as in mice treated with PD-1 blocking antibodies compared to the WT mice (91). These conflicting findings raise the caveat that PD-1’s role in the CNS can differ between viral infections and highlight the need for caution in extrapolating conclusions of immune responses across infection models. By extension, understanding how PD-1 controls T_RM_ development in different CNS viral infections should uncover novel insights in mechanisms of détente between viral control and collateral tissue injury by CD8 bT_RM_.

## Concluding Remarks

Accumulating evidence supports the concept that T_RM_ progenitors are generated early in the course of effector differentiation. An intriguing possibility is that factors such as TCR signal strength or differential expression of inhibitory receptors contributes to a nuanced differentiation spectrum that guides development of T_RM_. Similar ideas hold true for T_EX_. Recent work reveals that T_EX_ exist as a continuum from self-renewing “stem-like” progenitors that progress to a nonproliferative terminal state which is vulnerable to death. T_EX_ at different stages vary in their ability to respond to immune checkpoint blockade therapy (36). MuPyV-specific CD8 bT_RM_ heterogeneously express many molecules associated with T_EX_ subsets (36, 87). Single-cell analysis of adaptive immune cells in ulcerative colitis patients suggests that transcriptional heterogeneity also exists in the T_RM_ compartment and its demarcation into distinct differentiation stages (99). Similarly, lung CD8 T_RM_ generated to influenza infection exhibit both exhausted and memory characteristics by phenotype, transcriptome, and function (80). The proportion of T_RM_ in each stage of differentiation, however, will certainly be altered by disease processes and possibly by immunomodulatory regimens as well. Recent work also demonstrates that the quality of functional CD8 T_RM_ responses in the influenza-infected lung is dependent on the type of cell presenting viral antigens (100). Furthermore, T_RM_ can also egress from tissues, convert into other memory subsets, and change their migratory behavior depending on the inflammatory context (101, 102). Together these findings contribute to an increasingly multidimensional view of the factors that drive T_RM_ formation, what constitutes tissue residence, and the role T_RM_ play in antiviral defense. Particularly important for persistent neurotropic viruses is to develop a comprehensive understanding how bT_RM_ balance virus control against neuropathology and to learn how this equilibrium is established for different viral infections.

## Author Contributions

CN-W wrote the original draft and revised the manuscript. KA wrote the original draft, revised the manuscript, and prepared the figure. AL revised this manuscript. All authors contributed to the article and approved the submitted version.

## Funding

This work was funded by the National Institute of Neurological Disorders and Stroke and the National Cancer Institute grants R01NS088367 and R01NS092662 to AL, F32NS106730 to CN-W, and T32CA060395 to KA.

## Conflict of Interest

The authors declare that the research was conducted in the absence of any commercial or financial relationships that could be construed as a potential conflict of interest.

## Abbreviations

APC, antigen-presenting cell; bTRM, brain tissue-resident memory CD8 T cell; CNS, central nervous system; ColIV, collagen IV; FAO, fatty acid oxidation; i.c., intracranial; ICOS, inducible T-cell costimulator; IL, interleukin; ITIM, immunoreceptor tyrosine-based inhibition motif; ITSM, immunoreceptor tyrosine-based switch motif; Klf2, Kruppel-like factor 2; LCMV, lymphocytic choriomeningitis virus; MCMV, mouse cytomegalovirus; MHC, major histocompatibility complex; MPEC, memory precursor effector cell; MuPyV, mouse polyomavirus; PD-1, programmed cell death protein-1; PD-L1, programmed death-ligand 1; pMHC, peptide:MHC complex; PML, progressive multifocal leukoencephalopathy; S1P, sphingosine-1-phosphate; S1P1, sphingosine-1-phosphate receptor 1; SHP2, Src homology 2 domain-containing phosphatase 2; SLEC, short lived effector cell; T_CM_, central memory T cell; T_EM_, effector memory T cell; T_EX_, exhausted T cell; T_RM_, tissue-resident memory T cell; TCR, T cell receptor; VSV, vesicular stomatitis virus; WT, wild type.
